# Employment type and policy compliance with strict measures during public health crises: evidence from self-employed workers in China

**DOI:** 10.3389/fpubh.2025.1647494

**Published:** 2025-09-19

**Authors:** Tongzhou Lyu, Ruyi Yan, Yongxin Zhao, Jiangyin Wang, Xuke Zhang, Zhen Hong, Xiaozhu Huang

**Affiliations:** ^1^School of Politics and International Relations, East China Normal University, Shanghai, China; ^2^School of Philosophy, Zhongnan University of Economics and Law, Wuhan, Hubei, China; ^3^School of Humanities, Shanghai Jiao Tong University, Shanghai, China

**Keywords:** employment type, policy compliance, self-employed individuals, public health crisis, COVID-19

## Abstract

**Background:**

The rapid rise of non-standard types of employment—particularly self-employment—has significantly reshaped labor market structures and public policy systems. Compared to employees, self-employed individuals often lack long-term formal labor contracts and experience substantial income volatility, making them especially vulnerable. These vulnerabilities are further exacerbated during public health crises, adding complexity and uncertainty to policy implementation. This study examines the policy compliance of self-employed workers under strict public health measures, with the aim of exploring the relationship between employment type and policy compliance.

**Method:**

This study draws on 2,325 valid responses from the 2021 Chinese General Social Survey (CGSS2021). An Ordinary Least Squares (OLS) regression model is employed to compare levels of policy compliance between self-employed individuals and employees under strict public health measures. The analysis further examines how structural differences associated with employment type influence compliance behavior.

**Results:**

Self-employed individuals demonstrate significantly higher levels of policy compliance than employees during public health crises, with results statistically significant at the 1% level. Heterogeneity analysis further indicates that low-income self-employed individuals exhibit even greater compliance, also significant at the 1% level. In addition, self-employed workers in digitally advanced regions show higher compliance compared to their counterparts in less developed areas, with this difference likewise significant at the 1% level.

**Conclusion:**

Employment type is closely associated with policy compliance during public health crises. Challenging the common view that self-employed individuals are less compliant due to weak institutional constraints, this study finds they are more willing to comply. Motivation theory helps explain this pattern: normative pressures and external cues may heighten their sense of responsibility, driving normative compliance. While income instability, limited social protection, and regulatory uncertainty amplify risk awareness, driving calculative compliance. Heterogeneity analysis indicates that low-income self-employed individuals, driven by heightened risk perception and limited resilience, are more likely to comply with policies. Those in digitally advanced regions also exhibit higher compliance, possibly due to more efficient access to information, stronger industry governance, and more effective government oversight. This study contributes to a theoretical understanding of how employment type influences policy behavior, highlighting the distinct constraints and motivational foundations for policy implementation.

## 1 Introduction

Technological advancements have fundamentally reshaped work patterns and labor conditions, giving rise to increasingly diverse forms of employment type. Among them, self-employment, a major form of non-standard work, has expanded rapidly, restructuring labor markets and challenging traditional governance frameworks (1). Data from the International Labor Organization (ILO) and the World Bank indicate that the global number of non-standard workers rose from approximately 1.7 billion in 2005 to 2 billion in 2024, representing 58% of the global workforce (2). Among these, self-employed individuals number 1.62 billion, comprising 48% of the global workforce (3). Self-employment has become an integral component of modern labor markets, playing a crucial role in alleviating employment pressure and promoting economic dynamism (4). In the U.S., self-employed individuals grew from 9.6 to 11.8 million between 2020 and 2023, with an average annual growth of around 5%. Freelancers make up 36% of the U.S. workforce and contribute over one trillion dollars a year (5). China has also experienced rapid growth in self-employment. By 2023, ILO estimates show self-employed individuals made up 45.31% of China’s workforce (3). While self-employment has contributed to the global economy by expanding job options and increasing labor flexibility, it poses substantial institutional challenges. As a non-standard form of employment, self-employed workers often lack the protection of formal contracts, experience income instability, and are frequently excluded from social security systems (6). This large, decentralized, and highly flexible labor force is reshaping social governance and placing new demands on government capacity—particularly in areas such as labor regulation, social insurance coverage, tax collection and administration, and the maintenance of public order (7, 8).

The 2019 global outbreak of COVID-19 severely disrupted working conditions, household incomes, and basic livelihoods worldwide, pushing policy formulation and implementation into a more complex and uncertain context (9). During public health crises, high-intensity interventions penetrate daily life more directly than routine policies, often triggering dissatisfaction, resistance, or even backlash (10)—even when the public acknowledges their positive intent (11). Such reactions are particularly common among self-employed workers. Without stable income or institutional protection, they are under greater economic pressure and are more vulnerable to external shocks (12). They lack labor contract protections and have limited access to social security, leaving them at higher risk of livelihood loss and psychological stress under pandemic and policy pressure. Meanwhile, structural employment disruptions triggered by the pandemic further complicated the situation. Amid large-scale economic contraction and widespread job losses, many former employees were compelled to shift to self-employment, expanding this group continuously. The ILO estimates that between 2020 and 2022, around 1.6 billion informal workers—half the global labor force—faced direct risks of income loss. By 2023, self-employed workers constituted 47.3% of the global workforce, up from 45.8% in 2019 before the pandemic (13). These dynamics heightened both the vulnerability and complexity of self-employed individuals in policy execution, while also amplifying the institutional challenges governments faced in responding to this fragile group during the pandemic.

In general, public support, participation, and compliance are key to effective policy implementation—especially in emergency contexts (14, 15). The 2019 COVID-19 outbreak provided a valuable empirical lens to examine public behavior during policy enforcement. It also sparked increasing scholarly interest in understanding the mechanisms of citizen compliance during public crises. Many studies have explored the factors and mechanisms that influence citizen compliance with strict measures, including age, gender, education level, and ethnicity (16, 17). Against this backdrop, introducing employment type as an explanatory variable may offer new insights.

Research shows that employment types—such as formal jobs, self-employment, or flexible work—are linked to significant differences in psychological states like depression, sense of security, and wellbeing (18–20). These psychological traits, in turn, have been shown to correlate closely with policy compliance. For example, higher depression and lower wellbeing are significantly associated with less willingness to follow government measures (21). Furthermore, studies in sociology and labor economics suggest that employment status—whether steadily employed or unemployed—can indirectly shape compliance by affecting perceived risk, economic security, and political trust (22). This raises a valuable but yet under-explored question: Is there a correlation between differences in employment types and differences in policy compliance, and how are they related?

Based on this, this study draws on CGSS2021 data to examine how employment type relates to policy compliance, aiming to identify which groups are more likely to comply and to explore the potential explanatory mechanisms. Theoretically, this study makes two contributions. (1) It highlights employment type as a neglected dimension of social stratification in compliance research. Moving beyond demographic variables, it reveals how differences in resource access and risk response shape decisions, expanding the research perspective on social stratification and public policy interaction. (2) Building on existing literature, it uses employment type as an analytical lens to uncover unequal risk distribution and its feedback on policy outcomes, responding to concerns over differential compliance in risk society contexts. Practically, this study also makes two contributions. (1) By comparing compliance differences across employment types, it identifies “vulnerable groups” in policy implementation, helping ensure targeted support and guidance while offering theoretical insight for international crisis management. (2) As self-employment such as remote work and flexible labor are likely to persist in the long term, this study offers policy insight on balancing labor flexibility with social stability.

## 2 Literature review and research hypotheses

### 2.1 Employment types: self-employment and paid employment

Employment type refers to the way individuals engage in market-based production and economic activities. In 1993, the International Labor Organization (ILO) introduced the International Classification of Status in Employment (ICSE-93), which categorizes workers by legal status into two broad groups: paid employment and self-employment (23). Paid employees are those with a stable, ongoing contract—explicit (written or verbal) or implicit—with a single employer. Employers are responsible for tax withholding and social security contributions, under national labor regulations. In contrast, self-employment involves earnings directly linked to the profit (or potential profit) from goods or services produced, including self-consumption. Self-employed individuals operate independently, unaffiliated with organizations, and without long-term commitments to any employer. According to the OECD, self-employment encompasses employment of employers, own-account workers, members of cooperative producer society, and unpaid family labor (24).

Self-employed individuals and employees differ significantly in job characteristics, organizational culture, stress levels, and risk exposure. First, in terms of job characteristics, self-employed individuals lack formal contracts, enjoying greater autonomy and flexibility (25). In contrast, employees typically work under formal labor contracts and benefit from organizational support through institutional resources and working conditions. Their actions are also regulated by organizational rules and internal oversight. Second, regarding organizational culture and values, unlike the employed, self-employed workers are not constrained by a shared culture. Third, in terms of income source and economic pressure, employees depend mainly on wages and face no business-related risks. By contrast, self-employed workers rely on their goods or services for income, assume full risk, and often experience unstable earnings (26). Fourth, with respect to social security, self-employed individuals generally lack formal social protection due to the absence of employment contracts (27). These structural differences shape individuals’ experiences, mindsets, and social perceptions across two types, and become more evident during public crises, resulting in different coping abilities and behavioral responses.

### 2.2 Literature review

Existing research on the determinants of policy compliance has primarily focused on individual traits, policy design, target attributes, and contextual factors (14, 28). Studies suggest that enhancing citizens’ willingness to follow proactive preventive policies may be more critical than the policies themselves (29). During the COVID-19 pandemic, strict policies required citizens to adopt new behaviors and routines, often leading to disruption. Even when the benefits of such policies were recognized, individual-level factors still contributed to non-compliance (11). Compliance varied among groups with different sociodemographic characteristics (30), media exposure (31), risk perception (32), and political trust (33). The typo-logical classification of citizens has become a key method for studying how personal traits influence policy compliance (16, 34). However, limited attention has been given to how employment type affects policy compliance. The few existing studies on employment type tend to focus on specific occupations, highlighting how organizations function as social control mechanisms (35) that shape individual behavior through performance assessments and internalized cultural norms (22). Most compliance studies have concentrated on tax payment and information security (23). Therefore, investigating how employment type impacts compliance under strict public health measures is both timely and significant.

### 2.3 Research hypotheses

Self-employed individuals and employees differ notably in job characteristics, organizational culture, stress, and risk exposure. These differences may be association with policy compliance in several ways. First, autonomy and oversight vary. Employees are subject to organizational rules and internal supervision, making them more likely to comply with policies (36). By contrast, self-employed individuals’ autonomy and lack of supervision often result in compliance decisions based on self-interest (37). Second, organizational culture significantly influences behavior. For employees, norms of justice and morality often serve as internalized motivations for compliance. Organizational values, norms, and workplace atmosphere promote responsibility and order (38), thus reinforcing compliance. Self-employed workers, lacking such structures, rely more on personal judgment. Third, job stability and the transmission of psychological stress differ considerably. Compared to employees with formal organizational affiliations, self-employed workers face greater income volatility and stress (21), making them more susceptible to depression during crises (38). Prior studies have shown that low perceived security and depressive tendencies weaken compliance intentions (22). Fourth, limited social protection increases behavioral heterogeneity. Besides unstable employment, self-employed individuals often lack access to formal welfare systems. In crises, they bear greater risk alone, which undermines psychological security and lowers compliance motivation (39).

Public emergencies often involve strict enforcement that directly conflicts with individual interests (40). Under such conditions, differences in employment type may produce complex and compounded effects. The absence of organizational oversight and cultural regulation grants self-employed individuals greater behavioral flexibility; meanwhile, economic vulnerability amplifies psychological stress, and the lack of a social safety net diminishes confidence in the government’s ability to respond effectively.

Of course, in contrast to this line of interpretation, an alternative line of scholarship invokes the so-called “trust paradox.” Empirical evidence from early stages of the pandemic suggests that individuals—particularly those facing elevated infection risks—often experienced acute psychological ambivalence, in which heightened fear coexisted with skepticism or even denial. In such contexts, self-protective motives become especially salient, driving stronger support for containment behaviors (39, 41). Studies further show that the association between fear of the disease and endorsement of COVID-19 preventive measures is most pronounced when trust in government is low (42). Conversely, excessive confidence in governmental crisis management may attenuate personal protective motivation; when citizens perceive the state as the primary risk bearer, the sense of individual responsibility diminishes, leading to reduced voluntary compliance (43).

Building on these findings, we propose that the vulnerability and resource scarcity characteristic of the self-employed may amplify risk perceptions, motivating more proactive preventive behaviors. By contrast, employees embedded within formal organizations often receive institutional safeguards and support system, which may potentially diffuse individual responsibility to the firm or the state, thereby weaken their individual motivation to comply. A core objective of this study is to adjudicate between these competing explanations using Chinese data. Based on this, the following hypothesis was formed:

*H1:* Self-employed individuals exhibit lower levels of compliance during major public health crises.

However, this hypothesis may oversimplify the internal heterogeneity among the self-employed. Individual behavior is shaped not just by employment type, but also by the interaction of socioeconomic status and environmental conditions. These broader structural factors may reshape—or even reverse—the effects of employment type on individual behavior. In practice, the self-employed workers differ widely in terms of resources, financial conditions, and coping abilities. Income level is widely recognized as a key determinant of policy compliance. Research indicates that income groups exhibit distinct behavioral responses to major crises (44). According to the Health Belief Model, individuals facing a public crisis adjust their behavior based on their perceived risk to avoid harm. The greater the perceived severity, the more likely individuals are to adopt protective actions like mask-wearing or self-isolation (45). Within the self-employed workers, low-income individuals generally face greater survival pressure and structural disadvantages. Unlike higher-income self-employed workers (e.g., small business owners), groups like street vendors and gig workers often lack stable income, savings, and social protection, limiting their ability to withstand risks (46). On one hand, low-income self-employed workers face higher survival risks and precarious conditions—such as informal contracts, lack of remote work options, and limited workplace protections (47)—driving them to comply with health policies for self-protection. On the other hand, penalties like fines or forced closures may push low-income self-employed individuals into survival crises (48). Thus, out of self-interest, they tend to comply to avoid such consequences (42). Based on this, the following hypothesis was posited:

*H2a:* Low-income self-employed individuals exhibit higher levels of policy compliance.

Meanwhile, regional disparities in institutions and economic structures may also lead to differences in compliance. Recently, the digital economy, an emerging economic form, has rapidly evolved, reshaping local industries and employment patterns. The 2021 China Regional Digital Development Index Report highlights significant regional disparities, with the top 10 provinces labeled as digitally advanced (49, 50). These areas feature better digital infrastructure, wider mobile internet access, and rapid growth in gig, sharing, and platform-based employment (51). Digital platforms enable “deterritorialized” work, granting self-employed individuals greater flexibility in time, space, and resources—as seen among ride-hailing drivers, couriers, and livestream sellers (52). Therefore, digital development strongly correlates with the scale and activity of self-employment. Simultaneously, access to and engagement with health-related information are largely shaped by regional communication infrastructure (53). During the pandemic, an “information hunger” was widespread. In digitally advanced regions, self-employed individuals accessed health policy updates via social media, government alerts, and other platforms, which raised risk perception (54) and encouraged proactive compliance. These regions were also more likely to leverage technologies such as big data tracking, health codes, and digital payment records. Such tools improve enforcement by enabling real-time monitoring and traceable data, strengthening policy deterrence and precision. More importantly, digital technologies significantly enhance the monitoring and enforceability of policy implementation. For instance, platforms may algorithmically link behaviors like mask-wearing to job eligibility, compelling self-employed workers to comply.

In contrast, regions with weaker digital economies lack robust infrastructure, and self-employed workers tend to be traditional vendors dependent on local networks. In such contexts, regulatory agencies face challenges in delivering real-time and precise oversight, leading to policy implementation gaps and irregular behavior among self-employed individuals. Combined with intense local competition, such conditions often result in policy resistance or weak enforcement. Based on this, the following hypothesis was developed:

*H2b:* Self-employed individuals in digitally advanced regions show higher levels of policy compliance.

## 3 Methods and measurement

### 3.1 Data

This study uses data from the 2021 wave of the Chinese General Social Survey (CGSS). Conducted by the China Survey and Data Center at Renmin University of China, the CGSS systematically describes and analyzes Chinese society through annual surveys. It collects data across multiple levels—society, community, family, and individuals—and is regarded as one of China’s most authoritative longitudinal surveys. The dataset is known for its completeness, accuracy, and reliability. The data used in this study is the most recently released 14th wave (2021) of the CGSS, representing the latest available data since the outbreak of COVID-19 in 2019. It includes respondents’ reactions and attitudes, offering an updated snapshot of Chinese society during the pandemic. After accounting for all variables and removing missing or incomplete responses, 2,325 valid observations were retained for analysis.

### 3.2 Measurement

#### 3.2.1 Dependent variable

In this study, the dependent variable was defined as the degree of Chinese citizens’ compliance with the government’s COVID-19 prevention measures during the pandemic. Drawing on studies by Nivette and Folayan (17), the requirement set by the Chinese government for citizens to wear masks in public places was chosen as the indicator to measure their compliance level. This choice aimed to indirectly reflect citizens’ attitudes and compliance toward government epidemic prevention policies by observing their mask-wearing behaviors. The data for this variable comes from the 2021 CGSS questionnaire, specifically in response to the question: “During the peak of the epidemic, are you willing to comply with the government’s request of mask wearing?” For those who responded with “Certainly,” they were assigned a value of 4; “Might” was assigned a value of 3; “Might not” received a value of 2; and “Certainly not” was assigned a value of 1. Higher values represent greater levels of policy compliance.

#### 3.2.2 Independent variable

Building on existing literature (23, 55), this study aims to explore the relationship between employment type and citizens’ compliance with COVID-19 prevention policies. The independent variable is employment type, categorized into two groups: employed and self-employed individuals. This classification is based on the CGSS 2021 survey question: “Are you working within an organization?” Those who answered yes were coded as employees (0); those who answered no were coded as self-employed individuals (1).

#### 3.2.3 Control variables

Based on the research objectives and drawing on Wang et al. (78), the control variables include a range of individual-level characteristics: gender, religious belief, self-reported health status, vaccination status, ethnicity, educational attainment, marital status, political affiliation, psychological condition, household size, and social capital.

### 3.3 Model specification: OLS model

To explore whether different employment types are associated with variations in policy compliance, an Ordinary Least Squares (OLS) regression model is employed for preliminary estimation. The model is specified as follows:


$${\text{facemask}}_{i}=\alpha_{0}+\alpha_{₁}\cdot {\text{work}}_{i}+\alpha_{₂}\cdot X_{i}+\varepsilon_{i}$$

In this model, facemaskᵢ represents the level of policy compliance for respondent i; workᵢ denotes the employment type, specifically whether the respondent is self-employed; Xᵢ represents the set of control variables; and εᵢ is the error term. The coefficient *α*₁ captures the extent to which employment type affects policy compliance, and the significance of α₁ is the main focus of the analysis.

## 4 Results

### 4.1 Descriptive statistics

Table 1 reports the descriptive statistics.

**Table 1:** Descriptive statistics (*N* = 2,325).

Variable	Variable definition	Obs	Mean	Std. dev.	Min	Max
Facemask	Assign a value from 1 to 4, the higher the score, the higher the policy compliance	2,325	3.948	0.245	1	4
Organization	Employment = 0, self-employment = 1	2,325	0.323	0.468	0	1
Gender	Male = 1, female = 0	2,325	0.55	0.498	0	1
Religion	Religion = 1, no religion = 0	2,325	0.062	0.242	0	1
Nation	Han nationality = 1, others = 0	2,325	0.948	0.223	0	1
Education	Primary school and no education = 0, University degree or above = 1, Junior high school education = 2	2,325	1.382	0.685	0	2
Marriage	Married = 1, unmarried = 0	2,325	0.748	0.434	0	1
Political-status	CCP = 1, non-CCP = 0	2,325	0.154	0.361	0	1
Physical-condition	Assign a value from 1 to 5, the higher the score, the higher the self-rated health	2,325	2.126	0.859	1	5
Vaccine	Vaccine = 1, no vaccine = 0	2,325	0.837	0.369	0	1
Depression	Assign a value from 1 to 5, the higher the score, the higher the depression	2,325	1.876	0.946	1	5
Family	Assign a value from 1 to 4, the higher the score, the higher family member	2,325	2.303	1.628	0	17
Social_capital	Assign a value from 1 to 4, the higher the score, the lower the social capital	2,325	3.397	0.872	1	5
Job-income	Lower job income = 1, Middle job income = 2, Higher job income = 3	2,325	1.873	0.809	1	3
Region	Underdeveloped areas of digital economy = 1, Developed areas of digital economy = 2	2,325	1.548	0.498	1	2

In terms of policy compliance, the average score for compliance with the government’s mask-wearing requirement in public places during COVID-19 was 3.95. This indicates that, during the peak of the pandemic, most respondents strongly supported and complied with this preventive measure, given that mask-wearing was a primary method of epidemic prevention.

Regarding employment types, about 32% of respondents were self-employed workers, while 68% worked within organizations. This indicates that the majority were employed in organizational settings at that time.

With respect to individual characteristics, the gender distribution was fairly balanced, with 55% male and 45% female. Only 6.2% reported having a religious affiliation, suggesting that most respondents had no fixed religious belief. Educational levels were mainly concentrated at the junior and senior high school levels. The vaccination rate reached 83.7%, reflecting broad public acceptance. In addition, 74.8% were married, and 15.4% were Communist Party members. The average self-rated health score was 2.12, meaning most considered their health to be average. Meanwhile, the average depression score was 1.87, suggesting mild depressive symptoms in most cases.

As for social characteristics, the average household size was 2.30. In terms of social capital, the average frequency of meeting with friends was 3.40, indicating that most respondents socialized about once a month or less.

### 4.2 Benchmark regression analysis

Table 2 reports the results of the benchmark regression. Among them, Column (1) includes only the independent variable—whether a respondent is engaged in self-employment—to examine its relationship with the dependent variable, compliance with mask-wearing mandates. Column (2) adds controls for individual characteristics on the basis of Column (1); Column (3) incorporates household-related variables on the basis of Column (2); and Column (4) further includes social-level factors such as social capital, income level, and region on the basis of the preceding columns. The final interpretation is based on Column (4). After controlling for all relevant variables, the results show that self-employed individuals are more likely to agree that the government has the authority to mandate mask-wearing, and they show a higher willingness to comply. Specifically, their odds of compliance are 0.025 times higher than those of organizational employees, a difference significant at the 1% level. This finding contradicts Hypothesis 1, indicating the opposite of the original expectation.

**Table 2:** Benchmark regression results (*N* = 2,325).

Variables	(1)	(2)	(3)	(4)
	Facemask	Facemask	Facemask	Facemask
Organization	0.031***	0.033***	0.034***	0.030***
	(0.009)	(0.010)	(0.010)	(0.009)
Gender		−0.005	−0.007	−0.007
		(0.010)	(0.010)	(0.010)
Religion		0.001	0.000	−0.002
		(0.019)	(0.019)	(0.019)
Nation		−0.011	−0.012	−0.013
		(0.019)	(0.019)	(0.019)
Education		0.008	0.008	0.009
		(0.007)	(0.007)	(0.007)
Marriage		0.013	0.010	0.005
		(0.013)	(0.013)	(0.013)
Political-status		0.021	0.021	0.021
		(0.013)	(0.013)	(0.013)
Physical-condition			0.011	0.010
			(0.006)	(0.006)
Vaccine			−0.013**	−0.010
			(0.005)	(0.013)
Depression			−0.010	−0.013**
			(0.013)	(0.005)
Family				0.000
				(0.003)
Social_capital				0.008
				(0.006)
Province				0.002**
				(0.001)
_cons	3.938***	3.979***	3.942***	3.893***
	(0.007)	(0.042)	(0.031)	(0.040)
*N*	2,325	1,051	2,325	2,325
*R* ^2^	0.004	0.014	0.009	0.012

Robust standard errors are reported in parentheses. *, **, and *** indicate significance at the 10, 5, and 1% levels, respectively (applies throughout).

Among the control variables, demographic factors such as gender, ethnicity, education, and political affiliation show no significant association with compliance with mask-wearing mandates. However, psychological wellbeing matters. Respondents with fewer depressive symptoms were more likely to comply.

### 4.3 Robustness test

The previous results preliminarily verify that the self-employed individuals are more willing to comply with strict policies on wearing masks during public health crises compared to organizational employees. However, this finding requires further validation. To that end, we conduct two robustness tests.

The first test involves replacing the dependent variable. During the pandemic, governments implemented various containment policies beyond mask-wearing, such as restrictions on gatherings, business closures, and stay-at-home orders. These measures were closely tied to individuals’ work and daily routines. To capture broader dimensions of policy compliance, we use two alternative items from the 2021 CGSS survey: (1) “During the peak of the epidemic, are you willing to comply with the government’s request to shut down the workplace?” and (2) “At the peak of the pandemic, are you willing to comply with the government’s request to stay home?”

To ensure interpretability, responses were reverse-coded: For those who responded with “Certainly,” they were assigned a value of 4; “Might” was assigned a value of 3; “Might not” received a value of 2; and “Certainly not” was assigned a value of 1. Higher values indicate a stronger willingness to comply. Since both measures are continuous variables, we use OLS regression models for the robustness tests.

Table 3 reports the results. Column (1) presents findings based on the belief that the government can close businesses or workplaces. Column (2) shows results based on stay-at-home requirements. In both models, the effect of employment type on policy compliance remains consistently significant. These findings confirm that regardless of whether the policy concerns mask-wearing, workplace closure, or stay-at-home orders, self-employed individuals demonstrate a stronger tendency to comply with government mandates.

**Table 3:** Robustness check results for replacing dependent variable.

Variables	(1)	(2)
	Shut down the workplace	Stay home
Organization	0.078***	0.045***
	(0.017)	(0.016)
Control variables	Yes	Yes
_cons	3.713***	3.822***
	(0.070)	(0.060)
*N*	2,313	2,323
*R* ^2^	0.019	0.016

Second, we conduct another robustness test by redefining the independent variable. According to the ILO, a self-employed individual is someone who works in their own business or farm and does not receive a fixed wage or salary. This category includes employers, own-account workers, and contributing family workers. In the 2021 CGSS survey, employment type was measured not only by the question “Which of the following best describes your current employment situation?” but also by an additional item on the type of organization the respondent works for (including government agencies, enterprises, public institutions, social organizations, no affiliation, or the military). Based on the OECD definition, self-employed workers are individuals who work for themselves rather than being employed by an organization. In this context, those reporting “no organizational affiliation” are classified as self-employed individuals and coded as “1,” while all others are classified as employees and coded as “0.” Since the dependent variable remains continuous, we again apply an OLS regression model for this robustness check.

Table 4 presents the results. As shown, under the OECD-based classification of employment types, self-employed individuals continue to exhibit a higher level of policy compliance with mask-wearing than those employed in formal organizations. This finding is consistent with the results from the benchmark regression.

**Table 4:** Robustness check results of replacing independent variables.

Variables	(1)
	Facemask
Work units	0.030***
	(0.009)
Control variables	Yes
_cons	3.893***
	(0.040)
*N*	2,325
*R* ^2^	0.012

Third, we replace the estimation model. Given that the dependent variable is an ordered categorical measure with four levels (1 = “Certainly not,” 2 = “Might not,” 3 = “Might,”“4 = “Certainly “), we employ ordered logit and ordered probit models to honor the ordinal nature of the outcome and to assess the robustness of our findings. While OLS treats the ordered categories as continuous and implicitly assumes equal intervals between them, the ordered logit and probit models cumulative probabilities and explicitly respects the rank ordering of the categories.

Table 5 reports the results obtained after re-estimating the models. Column (1) presents the estimates from the ordered logit (ologit) model, and column (2) displays the ordered probit (oprobit) estimates. In both specifications, differences in employment status remain significantly associated with variations in policy compliance: the self-employed workers exhibit a markedly higher propensity to adhere to mask-wearing mandates.

**Table 5:** Robustness check results of the replacing estimation model.

Variables	(1) Ologit results	(2) Oprobit results
	facemask	facemask
Organization	0.644***	0.303***
	(0.238)	(0.102)
Control variables	Yes	Yes
cut 1	−6.277***	−2.810***
	(0.961)	(0.385)
cut 2	−5.428***	−2.536***
	(0.883)	(0.377)
cut 3	−2.618***	−1.434***
	(0.766)	(0.346)
*N*	2,325	2,325
*R* ^2^		

Last, to ensure the reliability of our estimates, we conducted a comprehensive set of diagnostic tests for the underlying assumptions of the original model. (i) Multicollinearity diagnostics reveal that all variance-inflation factors (VIFs) are below 2, with a mean of 1.07, indicating no serious collinearity (see Appendix Table A2). (ii) Because the model is relatively complex and non-linear interactions may induce heteroskedasticity, we reviewed the literature and found that White’s test is commonly preferred in comparable studies with large samples and few regressors (56). We therefore employ White’s test to enhance robustness. As shown in Appendix Table A3, White’s test yields *χ*^2^(97) = 90.91, *p* = 0.655, failing to reject the null hypothesis of homoskedasticity. (iii) Residual normality assessed via Jarque–Bera and Shapiro–Wilk tests indicates significant departures from normality. While OLS coefficient estimates remain unbiased, the reliability of *t*- and *F*-tests and associated confidence intervals is compromised. Accordingly, we re-estimated the model using robust standard errors and 1,000-replication bootstrapping. The results, presented in Appendix Table A4, columns (1) and (2), show that the bootstrap and robust standard errors are virtually identical, and the direction and significance of the key coefficients remain unchanged, confirming that our main conclusions are unaffected. Overall, the findings are robust across multiple diagnostic checks.

### 4.4 Heterogeneity analysis

Existing research suggests that differences in employment type can lead to variations in income, which in turn affect policy compliance. Even among those with the same type of employment, income disparities may result in differing levels of compliance. Moreover, regional economic development and sociocultural contexts influence both the distribution and nature of employment. In areas with more advanced digital economies, self-employed workers tend to operate more flexibly, and the extent to which they were affected by the pandemic also varies. To investigate this further, we examine whether the relationship between employment type and policy compliance differs across income groups and regions. Based on income classifications released by the National Bureau of Statistics (79), respondents are grouped into high-, middle-, and low-income categories. For regional classification, we rely on the Digital China Development Report (49) issued by the Cyberspace Administration of China, using the top 10 provinces with the most developed digital economies as digitally advanced regions, and all others as digitally less-developed regions. We then conduct OLS regressions within each subgroup. Results are presented in Table 6.

**Table 6:** Results of heterogeneity analysis.

Variables	(1)	(2)	(3)	(4)	(5)
	Low-job income group	Middle-income group	High-income group	Underdeveloped areas of digital economy	Developed areas of digital economy
Organization	0.048***	0.022	0.022	0.006	0.052***
	(0.017)	(0.019)	(0.019)	(0.013)	(0.018)
Gender	0.018	−0.023	−0.023	0.010	−0.018
	(0.017)	(0.018)	(0.018)	(0.012)	(0.016)
Religion	0.010	0.013	0.013	−0.017	0.018
	(0.033)	(0.036)	(0.036)	(0.024)	(0.034)
Nation	−0.042	0.047	0.047	−0.032	0.065
	(0.034)	(0.043)	(0.043)	(0.021)	(0.052)
Education	0.013	0.014	0.014	−0.004	0.017
	(0.010)	(0.014)	(0.014)	(0.009)	(0.012)
Marriage	0.012	0.014	0.014	0.016	−0.007
	(0.021)	(0.020)	(0.020)	(0.015)	(0.019)
Political-status	0.046*	0.037	0.037	0.003	0.034
	(0.026)	(0.026)	(0.026)	(0.017)	(0.023)
Physical-condition	0.003	0.009	0.009	0.015**	0.006
	(0.010)	(0.011)	(0.011)	(0.007)	(0.010)
Vaccine	−0.013	−0.027	−0.027	−0.013	−0.017
	(0.024)	(0.022)	(0.022)	(0.019)	(0.020)
Depression	−0.014	−0.009	−0.009	−0.016**	−0.011
	(0.009)	(0.010)	(0.010)	(0.007)	(0.009)
Family	−0.003	0.000	0.000	−0.002	0.003
	(0.005)	(0.005)	(0.005)	(0.004)	(0.005)
Social-capital	0.009	0.020*	0.020*	0.004	0.012
	(0.009)	(0.011)	(0.011)	(0.007)	(0.010)
_cons	3.950***	3.831***	3.831***	3.979***	3.816***
	(0.064)	(0.068)	(0.068)	(0.042)	(0.070)
*N*	926	768	768	1,051	1,274
*R* ^2^	0.022	0.020	0.020	0.014	0.015

Columns (1)–(3) show that in the low-income group, employment type has a positive and significant effect on policy compliance at the 1% level. This relationship is not observed in the middle- or high-income groups. These findings indicate that among low-income individuals, self-employed workers are more likely to comply with mask-wearing mandates, providing support for Hypothesis H2a. Columns (3) and (4) suggest that in digitally less-developed regions, employment type has no significant impact on compliance. However, in digitally advanced regions, self-employed workers are significantly more compliant, with results again significant at the 1% level. This confirms Hypothesis H2b.

## 5 Discussion

### 5.1 Differences in policy compliance across employment types

The empirical findings show that there is a significant correlation between employment form and policy compliance, even after controlling for individual factors such as age, education, and income. Surprisingly, the results contradict the initial assumption that self-employed individuals, due to weaker institutional embeddedness, would be less likely to comply. Instead, they showed higher levels of compliance than organizational employees during the public health crisis. This unexpected pattern may stem from differences in moral beliefs, risk perceptions, and motivational structures.

According to motivation theory, policy compliance can be understood through two distinct mechanisms (31, 57): normative motivation, which reflects individuals’ sense of moral duty, social obligation, and group identity (58), and calculative motivation, which is grounded in rational evaluations of costs and benefits (59). On one hand, self-employed individuals may demonstrate stronger compliance due to a combination of normative motivation and external normative constraints. On the other hand, given their lack of institutional support and higher exposure to livelihood risks, they may also comply strategically to avoid losses. These findings offer a more nuanced explanation of how employment type relates to policy behavior, underscoring the need to consider institutional position, economic vulnerability, and motivational pathways together.

#### 5.1.1 Normative motivation and policy compliance

From the normative motivation perspective, compliance is shaped by internal moral values and social norms, often reinforced by government trust (35, 60), a sense of social responsibility (56), interpersonal interaction (59), and peer pressure (58).

From an occupational perspective, conventional wisdom holds that self-employed individuals are less constrained by formal organizational rules and internal monitoring mechanisms, thus enjoying greater autonomy and showing lower levels of policy compliance. In contrast, employees are generally subject to institutional discipline and oversight, which should, in theory, promote compliance with government directives (61). However, this assumption may not fully apply in the context of a public health crisis.

One explanation lies in the “diffusion of responsibility” (62) effect. In organizational settings, responsibility is often dispersed across members, which can weaken individual accountability. If an organization fails to integrate public health mandates into its internal rules, employees may prioritize internal regulations over external policies. For example, in workplaces without clear COVID-19 protocols, even well-informed employees may neglect preventive measures due to performance evaluations or peer pressure. If mask-wearing is widely ignored in the office, employees may conform to group behavior rather than follow government guidance. Thus, the degree to which organizational norms align with public policy greatly shapes employees’ normative motivation. In contrast, although self-employed individuals operate outside formal institutions, their exposure to personal risk and the absence of organizational protection may amplify their motivation to comply. Without institutional buffers, self-employed workers are more likely to internalize government directives as risk-avoidance strategies.

Beyond this, a “trust paradox” may also influence policy compliance—namely, the counter-intuitive finding that higher political trust does not always translate into greater adherence to public policies. One manifestation of this paradox is “dependence-based trust.” Conventional wisdom suggests that strong political trust encourages citizens to follow government directives (54, 63). However, some studies have shown that excessive trust in the government’s capacity to manage a crisis may actually reduce individuals’ motivation to take protective actions on their own. When people assume that government measures are sufficient, they may feel less compelled to engage in additional precautionary behavior, thereby diminishing voluntary compliance (43). This tendency is particularly evident among organizational employees, who often benefit from institutional safeguards such as workplace guidelines and health resources. As a result, responsibility for pandemic response may be psychologically shifted onto the organization or government, leading to a relaxation of personal accountability. In contrast, self-employed individuals, who lack organizational support—including access to protective resources or reliable health information—are more likely to view government policies as their primary source of guidance. This greater dependence on external directives often results in stronger compliance with public health measures.

Another form is “compensatory compliance under low trust.” As prior research generally finds, declining confidence in the government tends to reduce public support for public policies (35, 57). While conventional wisdom suggests that declining trust in government leads to reduced support for public policies, some studies indicate that in low-trust environments, individuals may instead become more risk-aware and crisis-sensitive. This heightened sense of vulnerability can prompt them to adopt proactive protective behaviors as a way to compensate for perceived deficiencies in policy enforcement (48). For self-employed individuals, who often work in unstable and high-risk environments with little social security (40), the sense of exclusion and vulnerability is amplified during a crisis (64). As a result, they may comply more—not because they trust the system, but to compensate for its gaps. This is what we refer to as compensatory compliance.

#### 5.1.2 Calculative motivation and policy compliance

Under the framework of calculative motivation, policy compliance is understood as a rational decision-making process grounded in risk–benefit analysis. This perspective emphasizes that individuals, when confronted with external policy constraints or perceived threats, evaluate potential outcomes and choose the course of action that best serves their interests. Protection Motivation Theory (PMT) offers a useful lens for understanding this behavior. According to PMT, risk perception refers to a person’s subjective judgment of a specific threat’s characteristics and severity. It is often the first psychological response in a crisis (65). The perceived severity of risk and a person’s sense of vulnerability directly influence their willingness to adopt protective behavior (15). In this sense, PMT provides a valuable framework for understanding public policy compliance during a pandemic.

During public health crises, the economic vulnerability of self-employed workers can significantly strengthen the link between their policy compliance behavior based on calculative motivation. Unlike organizational employees, they lack institutional protections such as stable income, paid sick leave, and medical insurance. This makes them more susceptible to income and job loss (40). According to PMT, these elevated economic risks increase sensitivity to threats to personal health and safety, which may prompt more proactive protective behavior. In contrast, employees in formal organizations benefit from stronger labor contracts and social protection systems, which may lead them to underestimate the potential costs of health risks. Of course, a more granular classification of employees by organizational type would reveal that their compliance motives vary significantly across institutional contexts. Within highly regulated organizations, employees’ compliance behavior demonstrates markedly greater motivational complexity. For example, critical compliance actions among healthcare professionals—such as the mandatory disclosure of medical errors—are driven by a more intricate logic, deeply rooted in professional ethical norms, organizational culture, peer pressure, and the apprehension of severe occupational consequences, including license revocation (66). This provides a valuable comparative perspective for future research.

At the same time, those in unstable jobs with little to no social security tend to experience sharper declines in working hours and labor participation during crises (47). Factors such as income volatility, job uncertainty, and a lack of support networks are also closely linked to higher levels of depression and deteriorating mental health among self-employed workers (67). Research further suggests that elevated depressive symptoms and reduced psychological security can undermine both trust in government and willingness to comply with public policy (10, 68). However, in contexts of extreme resource scarcity, self-employed workers may still choose to comply—driven not by confidence in government, but by a fear of financial collapse. For them, compliance becomes a way to avoid uncontrollable risks (69).

### 5.2 Heterogeneity by personal income level and regional context

As shown in Table 5, there is significant heterogeneity in the association between employment types and policy compliance. Specifically, this difference is more pronounced among low-income individuals and in regions with more advanced digital economies, but much less evident among middle- and high-income groups or in less-developed digital regions. This finding suggests that the influence of employment type on individual behavior is not linear; rather, it is jointly shaped by economic resources and the digital environment. Possible explanatory pathways are discussed below.

First, low-income self-employed individuals tend to exhibit a stronger “survival rationality,” whereas their middle- and high-income counterparts are generally equipped with greater capacity to buffer risk. This difference results in divergent behavioral logics when it comes to policy compliance. Some studies have found that fear often drives individuals to submit to the protective authority of the state and to place their trust in the government’s ability to manage crises (70). During public health emergencies, low-income self-employed workers—such as street vendors and gig laborers—face heightened survival risks due to the absence of institutional protection, limited savings, and a lack of social network support (32, 47). This group is also disproportionately concentrated in high-contact service sectors, which are more susceptible to shutdowns and job losses (71). By contrast, middle- and high-income self-employed individuals—such as freelance lawyers and consultants—usually have a more stable economic foundation and are better positioned to absorb short-term policy shocks. Additionally, low-income individuals are less able to bear the financial consequences of noncompliance (72), such as fines and other penalties, which often impose severe burdens on those already struggling economically. As a result, low-income self-employed workers are more likely to internalize policy compliance as both a risk-avoidance mechanism and a component of their occupational ethics. For this group, following public health directives is not just about responding to state authority, but also a pragmatic survival strategy.

A second source of heterogeneity lies in regional differences. Self-employed workers in digitally advanced regions show higher compliance, which may be attributed to better access to information, stronger platform governance, and more effective government oversight. First, well-developed digital infrastructure improves access to timely and accurate information. Studies show that the content and frequency of media exposure shape how people perceive risk and decide whether to take protective actions (73). In areas with widespread internet coverage and fast information flows, self-employed workers are better informed and more responsive to policy updates, making them more likely to comply. Second, digital platforms—central to the digital economy—play a key role in regulating self-employed workers (74). Many platforms enforce hygiene and safety policies by embedding them in operational rules, with penalties for violations. These mechanisms help translate government requirements into professional norms, reducing the burden on formal regulation and strengthening compliance incentives among self-employed workers.

Third, governments in digitally advanced regions typically employ more sophisticated digital governance tools. With technologies like big data and cloud computing, they can monitor business activity in real time, improving both the precision and reach of enforcement. This form of technological deterrence increases pressure on self-employed workers to follow policy.

By contrast, in regions where the digital economy is underdeveloped, the difference in policy compliance between the self-employed workers and employees is statistically non-significant. This null finding likely reflects an “equalizing effect” arising from a dual vacuum of institutional supply and information. On the one hand, weak infrastructure constrains both the reach of government information campaigns and platform governance, generating a homogeneous information deficit that blunts behavioral distinctions tied to employment status (75). On the other hand, formal enforcement is also minimal: street-level inspections and community-grid monitoring—common in more developed areas—are largely absent (76). Consequently, the “platform algorithm plus state regulation” constraints that the self-employed workers might otherwise face fail to materialize, while employees are not subjected to stricter organizational oversight. As a result, both groups rely disproportionately on localized social networks for information and normative guidance, leading to statistically indistinguishable levels of compliance. In short, when state institutional supply is limited and digital governance tools are unavailable, employment status ceases to be a salient predictor of compliance; instead, the dominance of informal institutions—namely, kinship networks and community norms—neutralizes the “self-employed workers are more compliant” pattern observed in digitally advanced regions.

## 6 Conclusions and suggestion

### 6.1 Conclusion

Overall, the empirical findings deviate significantly from the initial hypothesis. In the context of a public health crisis, self-employed individuals demonstrated a higher level of policy compliance. This challenges the conventional view in existing literature, which suggests that self-employed individuals tend to show lower compliance due to the absence of organizational regulation and external oversight (38, 61).

Building on the structural differences across employment types, this study further explores the mechanisms underlying this counter-intuitive behavior. In terms of normative motivation, the vulnerability of the self-employed may lead them to rely more heavily on government information and policy guidance. At the same time, deteriorating personal circumstances may weaken their political trust, prompting them to support strict public health policies as a way to compensate for their own vulnerability (48). This offers additional empirical support for the existence of a “trust paradox” among self-employed groups. From the perspective of calculative motivation, the economic insecurity inherent in self-employment compels individuals to make rational decisions. They are more likely to comply with policies to reduce the threat to their livelihood and avoid potential penalties. In this sense, compliance becomes a strategy for minimizing risk. In contrast, organizational employees operate within formal regulatory systems. Their exposure to policy-related responsibilities and risks is often buffered or mediated by institutional hierarchies. As a result, both normative and calculative motivations may be weakened. These differences demonstrate how employment type shapes individual motivation structures and, in turn, influences behavioral responses during a public health crisis.

The structural inequalities observed during the COVID-19 pandemic are particularly pronounced in developing countries. Compared with middle- and high-income groups, low-income self-employed individuals experienced greater shocks, perceived higher risks, and had fewer resources to manage uncertainty. Consequently, they exhibited higher levels of policy compliance. Likewise, in regions with more developed digital economies, self-employed individuals were more willing to follow public health policies. This is likely due to better access to information, more structured industry governance, and stronger regulatory capacity. These findings highlight the heterogeneous constraints and motivational foundations underlying policy implementation across different groups and provide empirical insights into the dynamics of policy responsiveness.

### 6.2 Suggestions

First, policymakers and global health communicators should reject the stereotype that vulnerable groups are unwilling to comply with public policy. This study shows that low-income individuals—especially self-employed workers—often demonstrate higher compliance, driven by survival pressure and greater risk exposure. Emergency communication strategies should avoid stigmatization and instead adopt inclusive, empathetic messaging that acknowledges the lived realities of marginalized populations.

Second, more attention should be paid to the non-voluntary nature of compliance among vulnerable self-employed individuals. While their compliance rates may appear high, these behaviors are often shaped by fear of income loss and regulatory penalties, not genuine policy alignment. Governments and international aid institutions should provide stronger structural support—such as temporary subsidies, expanded social insurance, and minimum income protections—to enable more autonomous and sustainable compliance.

Third, investment in the digital economy should be prioritized to strengthen both communication and enforcement. This study finds that higher compliance in digitally advanced regions is driven by improved access to information, platform-level regulation, and the use of digital tools in government oversight. Scaling up digital infrastructure, encouraging platform-based governance, and promoting technological innovation can not only create new economic opportunities but also support public health systems. These tools enhance healthcare access, risk perception, and civic engagement—fostering a culture of proactive health behavior and improved policy compliance.

## 7 Limitations

Although this study sheds light on the differences in policy compliance between employment types, it still has several limitations. First, the classification of employment was broadly divided into self-employed workers and employees, without a more detailed breakdown of the self-employed group. This may overlook the internal structure and heterogeneity within self-employed individuals. Future research could adopt a more refined occupational classification to examine how different types of self-employments vary in motivational drivers and policy responses.

Second, the selection of proxy variables may have explanatory limitations. We use “mask-wearing behavior” as an indicator of policy compliance; although this action is highly policy-relevant and representative in the pandemic context, it captures only one facet of a multifaceted compliance construct. Prior studies have adopted similar indicators, underscoring their empirical feasibility, yet such measures inevitably fall short of encompassing the full spectrum of compliance behaviors (17, 77). Therefore, future research could consider adopting a more comprehensive measurement framework—such as combining responses to workplace closures, adherence to stay-at-home orders, and social-distancing practices—to construct a composite compliance index and thereby enhance overall explanatory capacity.

Third, the present study lacks direct measures and empirical tests of the mediating mechanisms—specifically, political trust and risk perception—that underpin our theoretical framework. Although these pathways are grounded in normative and calculative motivation perspectives, data constraints precluded comprehensive empirical validation. As a result, the discussion of these mediators remains theoretical, and the precise routes through which normative and calculative motives shape compliance await further validation. Future word could employ experimental designs or integrate mediators and moderators within a structural equation modeling framework, thereby constructing a more complete model, and clarify in the mechanisms linking employment type to policy compliance.

Finally, the study relies on cross-sectional survey data, which precludes a full depiction of the dynamic evolution of the self-employed worker’s compliance behavior. The findings therefore reflect only a statistical association between employment type and policy compliance, rather than causal relationship. In practice, the causal nexus is likely more complex: individuals who are inherently more compliant may self-select into self-employment, and unobserved characteristics—such as risk-coping capacity or social capital—may simultaneously affect both employment choice and compliance behavior, resulting in residual confounding. Accordingly, all inferences in the manuscript are framed in terms of association rather than causation. Future research can leverage panel data and employ fixed- or random-effects estimators to capture within-individual variation in compliance across successive policy shocks or epidemic phases, thereby tracing the temporal trajectories of adherence among different employment groups. In parallel, randomized controlled trials (RCTs) or natural experiments can be designed to exogenously manipulate the intensity of normative and calculative motivations, enabling direct causal identification of their respective effects on compliance. Such designs will provide a more comprehensive understanding of the policy-response dynamics and behavioral evolution mechanisms characterizing the self-employed during public crises.

## Data Availability

The datasets presented in this study can be found in online repositories. The names of the repository/repositories and accession number(s) can be found below: http://www.cnsda.org/index.php?r=projects/view&id=65635422.
