# Supplementary material for: Employment type and policy compliance with strict measures during public health crises: evidence from self-employed workers in China
Source: Front Public Health. 2025 Sep 19;13:1647494. doi: 10.3389/fpubh.2025.1647494 (PMC12491208; doi:10.3389/fpubh.2025.1647494)
Supplement: Supplementary file 1 [file Data_Sheet_1.docx]

**Appendix**

Appendix A1 CGSS 2021 Variable Description

| **Variable name** | **CGSS2021 variable code** | **Original item** | **Answer options** | **Data cleaning/coding rules** |
| --- | --- | --- | --- | --- |
| facemask | D27_d | "During the peak of the epidemic, are you willing to comply with the government's request of mask wearing?" | 4=“Certainly”;  3=“Might”;  2="Might not";  1="Certainly not". | reverse encoding |
| organization | A59j | What is the type of unit or company you are currently working for? | 1 = Party and government agencies;  2 = Enterprises;  3 = Public institutions;  4 = social organizations,  neighborhood/village committees;  5 = No affiliation / self-employed (including individual proprietors);  6 = Military | Mark 5 as 1 (self-employed workers), and mark the rest as 0 (employee). |
| gender | A2 | Gender | 1=man  2=woman | man=1  woman=0 |
| religion | A5 | What is your religious belief? | 1=not religious  10< religious | Religion belief recorded as 0, non-religion belief recorded as 1 |
| nation | A4 | What is your ethnicity? | 1=Han nationality;  2=Mongolian nationality;  ...  7=Uyghur nationality. | Encode as 0 for Han nationality, and mark the rest as 1. |
| education | A7a | What is your current highest level of education? | 1 = No formal education received;  2 = Private tutoring, literacy class;  3 = Primary school;  4 = Junior high school;  ...  12 = Bachelor's degree (formal higher education);  13 = Postgraduate degree or above. | 0 represents primary school or higher education, 1 represents college or higher education, 2 represents junior high school or high school education. |
| marriage | A69 | What is your current marital status? | 1 = unmarried;  2 = Cohabiting;  3 = First marriage with spouse;  ...  6 = Divorced;  7 = Widowed. | 1, 2, 6, and 7 are coded as 0 (single), while 3, 4, and 5 are coded as 1 (married). |
| political-status | A10 | What is the current political situation? | 1=the general public;  2=League of Communist Youth member;  3=democratic parties;  4=Communist Party member. | Mark as 1 for Communist Party members, and mark as 0 for non-Communist Party members. |
| physical- condition | A15 | What do you think is your current physical health condition? | 1 = Very unhealthy;  2 = Unhealthy;  3 = Average health;  4 = Relatively healthy;  5 = Very healthy. | Reverse encoding |
| vaccine | V1 | At present, have you received the COVID-19 vaccine? | 1=has been vaccinated  2=has not been vaccinated | Mark as 0 (not vaccinated) |
| depression | A17 | How frequently have you felt depressed or discouraged in the past four weeks? | 1=always;  2=often;  3=sometime;  4=seldom;  5=never. | reverse encoding |
| family | A1 | Besides you, how many other people are there in your family? | \ | Utilize raw data |
| social_capital | A30_7 | Have you often gathered with friends during your free time in the past year? | 1 = Daily;  2 = Several times a week;  3 = Several times a month;  4 = Several times a year or less;  5 = Never. | Utilize raw data |
| Province | s41 | Interview location - province/autonomous region/municipality code | \ | Utilize raw data |
| job-income | A8b | What was your personal total income for the whole year of last year (2020)? | \ | “>95836” marked as 3  “<44949” marked as 1  The group between the two extremes is marked as 2. |
| region | s41 | Interview location - province/autonomous region/municipality code | \ | Regions with advanced digital economies are marked as 2, while other regions are marked as 1. |

Appendix A2 VIF and 1/VIF Statistics

| Variable | VIF | 1/VIF |
| --- | --- | --- |
| physical condition | 1.15 | 0.872588 |
| depression | 1.12 | 0.889549 |
| marriage | 1.12 | 0.889798 |
| organization | 1.08 | 0.923999 |
| politicals~s | 1.08 | 0.927608 |
| family | 1.08 | 0.928168 |
| social_cap~l | 1.07 | 0.933086 |
| religion | 1.04 | 0.962512 |
| education | 1.04 | 0.962759 |
| nation | 1.03 | 0.966202 |
| gender | 1.03 | 0.967769 |
| vaccine | 1.02 | 0.978191 |
| province | 1.02 | 0.983462 |
| Mean VIF | 1.07 |  |

Appendix A3 White tests for heteroskedasticity

| Test Type | Chi-Square Value | Degrees of Freedom | p-Value |
| --- | --- | --- | --- |
| Heteroskedasticity | 90.91 | 97 | 0.6549 |
| Skewness | 35.64 | 13 | 0.0007 |
| Kurtosis | 7.11 | 1 | 0.0077 |
| Total | 133.66 | 111 | 0.0705 |

Appendix A4 Robust standard error result

|  | 1. Robust standard error result | （2）bootstrap  Result |
| --- | --- | --- |
|  | facemask | facemask |
| organization | 0.030*** | 0.030*** |
|  | (0.009) | (0.009) |
| gender | -0.007 | -0.007 |
|  | (0.010) | (0.011) |
| religion | -0.002 | -0.002 |
|  | (0.019) | (0.020) |
| nation | -0.013 | -0.013 |
|  | (0.019) | (0.020) |
| education | 0.009 | 0.009 |
|  | (0.007) | (0.007) |
| marriage | 0.005 | 0.005 |
|  | (0.013) | (0.014) |
| politicalstatus | 0.021 | 0.021 |
|  | (0.013) | (0.014) |
| SFH1 | 0.010 | 0.010 |
|  | (0.006) | (0.007) |
| vaccine | -0.010 | -0.010 |
|  | (0.013) | (0.013) |
| depression | -0.013** | -0.013** |
|  | (0.005) | (0.005) |
| family | 0.000 | 0.000 |
|  | (0.003) | (0.003) |
| social_capital | 0.008 | 0.008 |
|  | (0.006) | (0.006) |
| province | 0.002** | 0.002** |
|  | (0.001) | (0.001) |
| _cons | 3.893*** | 3.893*** |
|  | (0.040) | (0.040) |
| N | 2325 | 2325 |
| R^2^ | 0.012 | 0.012 |
